# Special Hospitals and Their Visiting Staffs—A Disclaimer

**Published:** 1907-06-22

**Authors:** John MacKinna

**Affiliations:** Secretary. The Metropolitan Ear, Nose, and Throat Hospital, Grafton Street, Tottenham Court Road, W.


### SPECIAL HOSPITALS AND THEIR VISITING STAFFS—A DISCLAIMER.

Dear Sir,—With reference to the article bearing the above title and appearing in your issue of June 15, page 274, I shall be obliged by your kindly allowing me space to refute on behalf of the members of the staff of the Metropolitan Ear, Nose, and Throat Hospital the statements made by Mr. Francis Roe, in justice to this, if not the first, one of the oldest of special hospitals for diseases of the ear, nose, and throat. I trust that you will give to this disclaimer the same publicity which you have rightly given to the episode related by Mr. Francis Roe.

I am, yours faithfully,
John MacKinna, Secretary.

The Metropolitan Ear, Nose, and Throat Hospital, Grafton Street, Tottenham Court Road, W.

